# Specific and Non-Invasive Fluorescent Labelling of Extracellular Vesicles for Evaluation of Intracellular Processing by Intestinal Epithelial Cells

**DOI:** 10.3390/biomedicines8070211

**Published:** 2020-07-14

**Authors:** Maria S. Hansen, Ida S. E. Gadegaard, Eva C. Arnspang, Kristine Blans, Lene N. Nejsum, Jan T. Rasmussen

**Affiliations:** 1 Department of Molecular Biology and Genetics, Aarhus University, 8000 Aarhus, Denmark; Ida-gadegaard@hotmail.com (I.S.E.G.); krbla@arlafoods.com (K.B.); 2 Department of Clinical Medicine, Aarhus University, 8000 Aarhus, Denmark; arnspang@kbm.sdu.dk (E.C.A.); nejsum@clin.au.dk (L.N.N.)

**Keywords:** milk, extracellular vesicles, exosomes, labelling, lactadherin, fluorescence, uptake, intestinal epithelium, Caco-2, endosomes

## Abstract

The presence of extracellular vesicles (EVs) in milk has gained interest due to their capacity to modulate the infant’s intestinal and immune system. Studies suggest that milk EVs are enriched in immune-modulating proteins and miRNA, highlighting their possible health benefits to infants. To assess uptake of milk EVs by intestinal epithelial cells, a method was developed using labelling of isolated EVs with fluorophore-conjugated lactadherin. Lactadherin is a generic and validated EV marker, which enables an effective labelling of phosphatidylserine (PS) exposing EVs. Labelled EVs could effectively be used to describe a dose- and time-dependent uptake into the intestinal epithelial Caco-2 cell line. Additionally, fluorescence microscopy was employed to show that EVs colocalize with endosomal markers and lysosomes, indicating that EVs are taken up via general endocytotic mechanisms. Collectively, a method to specifically label isolated EVs is presented and employed to study the uptake of milk EVs by intestinal epithelial cells.

## 1. Introduction

Extracellular vesicles (EVs) are membrane-surrounded vesicles released into the extracellular space by cells. The term EV encompasses a broad and heterogeneous population of secreted vesicles, including exosomes (30–120 nm), microvesicles (50–1000 nm), and apoptotic bodies (500–2000 nm), which broadly differ in size, content, and biogenesis. Over the past years, EVs have been recognised as important vehicles for intercellular communication. This kind of vesicle has been isolated from many different tissues, healthy and diseased, and every biological fluid in the body, including saliva, blood, urine, and milk. The soluble core of EVs contains mRNA and microRNA (miRNA) with the potential to modulate the recipient cell function [1]. These shed membrane vesicles are therefore suggested to have various biological functions and potentially also as natural carriers for drug delivery [2].

Milk is a highly complex, variable biological fluid and important source of infant nutrition. It has evolved over many years to provide the best nourishment for infants, and protects from diseases during the first months of life, while the immune system matures [3].

EVs have been identified in human [4,5,6,7], bovine [7,8,9], sheep [10], camel [11], and porcine milk [12]. Additionally, commercial, processed bovine milk has been shown to contain intact EVs with a content of miRNA [13]. Breast milk EVs regulate immune cell responses [4,14], and the cargo is dynamic with an enrichment of immune related proteins and miRNA in colostrum EVs compared to mature milk EVs [4,9,15,16].

Milk-derived EVs are taken up by various cultured cells within and across species, e.g., macrophages [5,16,17], lung cancer cells [18], endothelial cells [19,20], and intestinal epithelial cells [21]. These studies, however, primarily employ lipophilic fluorescent dyes. Additionally, milk EVs are investigated for their therapeutic potential as a vehicle for transmitting molecules to tissues, either admitted orally or intravenously, due to their natural and nontoxic nature [22].

The interest in EVs rises due to their potential capacity to induce phenotypic changes in target cells. This ability to communicate with the surroundings makes EVs subjects of research within fields of disease and stem cell biology, both of which are topics of extensive research. The biology behind vesicle budding, intracellular exosome formation, EV secretion, and EV uptake mechanisms has been investigated and described in various publications and reviews [23,24,25]. Additionally, many reports assess physiological responses in cells induced by EVs, but information is sparse on intracellular trafficking and sorting of EV material after uptake.

EVs communicate with acceptor cells via different mechanisms, e.g., by activating extracellular receptors and thereby only acting at the cell membrane [26]. However, the unique and characteristic feature of EVs is the enclosed lipid bilayer with cargo safely protected inside. It does therefore seem likely that EVs fuse with the plasma membrane or are endocytosed/internalized to deliver their cargo to the intracellular space. Most studies investigating mechanisms for EV uptake report on some sort of endocytosis [25,27,28,29], but fusion with the plasma membrane has also been suggested [30]. A valid explanation for lack of consensus on this matter is that uptake is cell type and EV origin specific and probably also depends on the size of the EV [31].

After cell internalization, it has been reported that T-cell derived EVs colocalized with both LAMP1 and Rab7 (lysosomal-associated protein and late endosomes, respectively) in macrophages [27]. Additionally, tumour cell-derived EVs colocalized with lysosomes and early endosomes, but not ER, in the mother cell line [32]. Furthermore, and perhaps unexpected, studies found that EVs were delivered to the nucleus of recipient cells [33], and to the ER before ending up in lysosomes as the final destination [34]. The latter two studies reported on intracellular EV trafficking via the endosomal system.

Lipophilic fluorescent dyes, such as PHK26 and PKH67, are often used to stain EVs to follow uptake and intracellular processing. However, a study by Morales-Kanstresana (2017) elegantly shows how the amphiphilic nature of these dyes lead to micelle formation in sizes ranging from 100–400 nm, thereby overlapping with EVs [35]. Moreover, issues and pitfalls regarding the use of lipophilic dyes to stain isolated EVs have been addressed elsewhere [36]. Among the issues are lack of specificity of labelling and difficulties in separating excess dye from EVs.

To overcome the use of lipophilic dyes, the glycoprotein lactadherin conjugated to a fluorophore is used to fluorescently label EVs. Labelled lactadherin has previously been used to label exosomes secreted by transfected cells to follow their biodistribution [37]. Lactadherin is a membrane associated glycoprotein, which binds phosphatidylserine tightly in phospholipid bilayer membranes via the C-terminal C2 domain [38,39]. Lactadherin is accepted as a general EV marker [40,41,42] and its association with both human and bovine milk EVs has been identified in mass spectrometric analyses and Western blots in relatively high concentrations [4,7,8].

The primary aim of the present study was to develop a method to fluorescently label isolated EVs with the naturally present protein lactadherin, and thereby avoiding introduction of artificial substances such as lipophilic dyes. Fluorescently labelled EVs provide the basis for studying how EVs are taken up in vitro by intestinal epithelial cells and sorted intracellularly in the endosomal system. Finally, evidence is provided that emphasizes the importance of a proper cell wash for removal of non-internalized EVs from the cell surface, especially when studying uptake quantitatively.

## 2. Experimental Section

### 2.1. Milk EV Isolation and Concentration

The starting material for milk EV isolation was fresh, unprocessed bovine tank milk collected less than 48 h after milking. The isolation procedure was performed as described by [7]. Briefly, fresh, raw bovine tank milk was supplemented with 0.5 µM of the protease inhibitor phenylmethane sulfonyl fluoride and centrifuged at 3400× *g* for 35 min at 4 °C to pellet cells and subsequently removal of the firm cream layer. The resulting skimmed milk was then subjected to another centrifugation at 20,000× *g* for 60 min at 4 °C to sediment main casein components. Casein reduced clear milk serum was drawn from the centrifugation tubes and 25 mL was subjected to size exclusion chromatography (SEC) on a 2.5 × 88 cm (432 mL) Sephacryl S−500 column in 0.2 µM filtered PBS with EDTA (50 mM NaH_2_PO_4_, 0.15 M NaCl, 20 mM EDTA) pH 7.4. Absorbance at 280 nm was measured on each resulting SEC fraction to identify light scattering particle- and protein-containing fractions. Fractions shown to contain EVs were pooled. To obtain a more concentrated EV fraction, the pooled EVs were subjected to reverse osmosis dialysis in a dialysis tube (cut off value 12–14 kDa) (Medicell International Ltd., London, UK) against 10% polyethylene glycol 20,000 (*w*/*v*) dissolved in PBS buffer, pH 7.4, overnight at 4 °C while stirring.

### 2.2. Quantification of EV Protein Concentration

EV quantification was based on protein concentration in the isolated EVs. Protein concentration was estimated by a modified Lowry method [43,44]. A serial dilution of bovine serum albumin (BSA) (Sigma Aldrich, St. Louis, MO, USA) served as the standard curve. Dilutions of EVs and BSA was subjected to the following treatment: 225 μL sample was mixed with 25 μL 10% SDS and 250 μL alkaline Cu-reagent (50 g Na_2_CO_3_, 0.5 g potassium tartrate, 0.25 g CuSO4, dissolved in H_2_O) and incubated 10 min at room temperature, before 1 mL phenol reagent (2.78 mL Folin Ciocalteau phenol in 50 mL H_2_O) was added and samples were heated at 55 °C for 5 min. Next followed a quick cooling by putting samples on ice. In triplicates, absorption was measured spectrophotometrically in all samples at 630 nm and averaged.

### 2.3. Western Blotting

Two µg protein from the indicated fractions was separated on 10% BisTris NuPAGE (Thermo Fischer Scientific, Hillsboro, OR, USA) using MOPS buffer followed by transfer to polyvinylidene fluoride membranes (500 V, 200 mA, 1 h). Membranes were blocked in 2% Tween−20 in TBS prior to probing with primary antibodies. Following antibodies were used: Mouse anti-human CD81 (clone: 12C4) (Cosmo Bio, Tokyo, Japan) and in-house produced rabbit anti-bovine lactadherin.

### 2.4. Lactadherin–Fluorophore Conjugation

Bovine lactadherin was conjugated to Alexa Fluor 488 or Alexa Fluor 568 NHS (N-hydroxysuccinimide) (Thermo Fischer Scientific, Hillsboro, OR, USA) according to the manufacturer’s protocol in an experimentally determined molar ratio of 13:1 (dye to protein). Excess dye was removed by dialysis (cut off value 12–14 kDa) placed in PBS under stirring over two days at 4 °C in the dark. Alexa Fluor-conjugated lactadherin was stored at −80 °C until further use. Degree of labelling was determined according to manufacturer’s protocol.

### 2.5. EV Labelling

Purified and concentrated EVs incubated with 200 nM lactadherin-Alexa Fluor for 24 h at 37 °C. Afterwards, excess, unbound lactadherin–Alexa Fluor was separated from the EVs by SEC using a 0.8 × 20 cm (10 mL) Sephacryl S−500 column in 0.2 µM filtered PBS (50 mM NaH_2_PO_4_, 0.15 M NaCl) pH 7.4 (flow: 12 mL/h, fraction size 1 mL). Absorbance at 280 nm was measured on the resulting fractions to indicate the fractions containing EVs. The presence of fluorophore-conjugated lactadherin was determined in each fraction using a microplate reader (PerkinElmer, Waltham, MA, USA) with excitation/emission wavelengths at 490/525 nm. Moreover, the presence of EVs was confirmed by confocal microscopy on selected fractions. The fractions containing labelled EVs were pooled and protein concentration determined according to a standard curve based on protein concentration correlated with absorbance at 280 nm.

### 2.6. Size Distribution Measurements

Size distribution was determined on EVs within 24 h after purification and prior to concentration and on EVs after labelling using the NanoSight LM10 system (Nanosight Ltd., Amesbury, UK). The two samples were diluted 1000× in particle-free PBS (50 mM NaH_2_PO_4_, 0.15 M NaCl). Cameral level was kept at 12 and detection threshold at 11. Five successive measurements were made, temperature manually entered, and results averaged to obtain size profiles.

### 2.7. Caco-2 Cell Culture Maintenance

The human adenocarcinoma cell line Caco-2 cells was obtained from German Collection of Microorganisms and Cell Cultures GmbH (DSMZ) (ACC 169). Cells were cultured in DMEM medium (Dulbecco’s modified eagle medium) (Gibco, Thermo Fischer Scientific, Hillsboro, OR, USA) containing Glutamax, glucose (4.5 g/L), pyruvate, and supplemented with 10% (*v*/*v*) foetal bovine serum (*v*/*v*), and 1% (*v*/*v*) penicillin/streptomycin (Gibco, Thermo Fischer Scientific, Hillsboro, OR, USA). Cells were maintained at 37 °C and 5% CO_2_ in a humidified chamber and used from passage 13–40 in the present study.

### 2.8. Quantitative Uptake Experiments

Caco-2 cells were grown in 96-well plates (Costar 3628, Thermo Fischer Scientific, Hillsboro, OR, USA) to 95–100% confluency and serum-starved 24 h prior to stimulation with labelled EVs in serum-free medium. Following EV incubation at indicated EV concentrations and time points, medium was removed and cells washed three times with citrate buffer followed by a neutralizing wash in PBS buffer before lysis with 1% Triton X-100 in PBS buffer. Total fluorescence in each well was determined using a microplate reader (PerkinElmer, Waltham, MA, USA). Excitation wavelengths were at 490 nm (Alexa Fluor 488, Thermo Fischer Scientific, Hillsboro, OR, USA), 510 nm Octadecyl Rhodamine B Chloride (R18), or 578 nm (Alexa Fluor 568) and emission wavelengths were measured at 525 nm, 545 nm, or 603 nm respectively.

### 2.9. MTT Assay

Caco-2 cells were seeded in 96-well plates at a density of 5000 cells/well and grown for three days. Thereafter, medium was replaced, and cells incubated in the absence (control) or presence of the indicated concentration of EVs for 3, 6, or 24 h. The MTT (3-(4,5-dimethylthiazol-2-yl)-2,5-diphenyltetrazolium bromide) assay was conducted according to Thermo Fisher Scientific’s protocol (Vybrant^®^ MTT Cell Proliferation Assay Kit). Briefly, media was discarded followed by a single cell wash. PBS was then replaced with fresh phenol red-free media (Dulbecco’s Modified Eagle’s Medium; 21063–029; Gibco, Thermo Fischer Scientific, Hillsboro, OR, USA) and 10 μL of a 12 mM MTT-solution was added to every well. Cells were incubated for 2 h at 37 °C. Afterwards, media was discarded, and dimethyl sulfoxide was added to dissolve the formazan crystals. By measuring the absorbance at 540 nm (EnSpire Multimode Plate Reader; PerkinElmer, Waltham, MA, USA), the amount of metabolic active cells was quantified and compared to the control.

### 2.10. Cell Wash

The efficacy of different washing buffers in washing non-internalized EVs away from cell membranes was tested with the following buffers: PBS buffer (50 mM NaH_2_PO_4_, 0.15 M NaCl, pH 7.4; glycine buffer (0.1 M glycine, pH 2.5); and citrate buffer (10 mM sodium citrate, 0.05% Tween 20, pH 6.0). All buffers were filtered with a 0.22 µm filter before use. Cells were grown to 95–100% confluency and serum-starved 24 h prior to experiment, then stimulated with 30 µg/mL labelled EVs in serum-free medium for two hours before a triple wash in the specified buffer followed by 1× PBS buffer wash to neutralize pH.

### 2.11. Constructs, Transfection, and LysoTracker

Plasmids used for transfection included Rab5-GFP, Rab7-GFP, and Rab11-mCherry (obtained from Addgene, Watertown, MA, USA). Lipofectamine 2000 (life technologies) served as transfection agent. For each transfection, 6 µL Lipofectamine was mixed with 500 µL Opti-MEM medium (Gibco, Thermo Fischer Scientific, Hillsboro, OR, USA) plus 2 µg DNA. A density of 200,000 Caco-2 cells in suspension were seeded in DMEM medium supplemented with 10% foetal bovine serum (FBS) and 1% penicillin/streptomycin followed by the addition of 500 µL transfection mixture. Medium was replaced with fresh medium after four hours. Fluorescence microscopy was performed 48 h post transfection. Lysosomes were stained with LysoTracker Red (life technologies, Thermo Fischer Scientific, Hillsboro, OR, USA) according to manufacturer’s protocol. Briefly, 50 nM LysoTracker Red was diluted in serum-free medium and added to the cells for 15 min followed by a PBS wash.

### 2.12. Fluorescence Microscopy

For microscopy, cells were grown on coverslips coated with Cultrex Basement Membrane Extract (Trevigen, Gaithersburg, MD, USA). After EV stimulation and cell wash with citrate buffer, cells were fixed in 4% paraformaldehyde for 15 min. Nuclei were stained with 2 µg/mL 4′-6-diamidino-2-phenylindol (DAPI) (Thermo Fischer Scientific), and in one experiment cell membranes were stained with 68 nM Octadecyl Rhodamine B Chloride (R18) (Invitrogen, Thermo Fischer Scientific, Hillsboro, OR, USA) prior to fixation. Fixed Caco-2 cells were imaged on a Nicon Eclipse Ti-E microscope equipped with a 100× oil objective, a Zyla 5.5 sCMOS camera (Andor Technology Ltd., Belfast, UK) and a pE-300white LED unit (CoolLED, Ramcon A/S, Birkerød, Denmark) and filtercubes for DAPI, EGFP, and TexasRed. Different fluorophores were imaged sequentially and merged afterwards. Light intensities were adjusted individually, and kept constant, for each sample and comparison.

Image processing and analysis was performed in Fiji (ImageJ, U.S. National Institutes of Health, Bethesda, MD, USA) [45].

### 2.13. Statistical Analysis

A one-way ANOVA was used to compare the efficacy of different wash buffers in washing off non-internalized EVs from plasma membranes and to analyse the effect of EV concentration on cell viability for three different incubation times. Specific differences were investigated using Tukey’s multiple comparisons test. Model validation included tests for equal standard deviations and normal distribution. All statistical tests were performed with GraphPad Prism version 7.0 (GraphPad Software, La Jolla, CA, USA) with an alpha level of *p* < 0.05. All data are shown as means ± SD.

## 3. Results

### 3.1. Labelling of EVs Using Lactadherin Conjugated to a Fluorescent Tag

Fresh, raw, bovine milk constituted the starting material for EV isolation. The isolation procedure followed the ‘serum isolation’ as described previously [7]. This method is characterized by being a gentle, pellet-free milk EV enrichment method. Initially, two centrifugation steps separate milk fat globules, milk cells, and the majority of micellar casein from the whey fraction. The supernatant containing the remaining part of the milk phospholipid is then subjected to size exclusion chromatography (SEC), which ultimately separates the large and globular EVs from remnants of milk protein (see Supplementary Figure S1).

**Figure 1:**
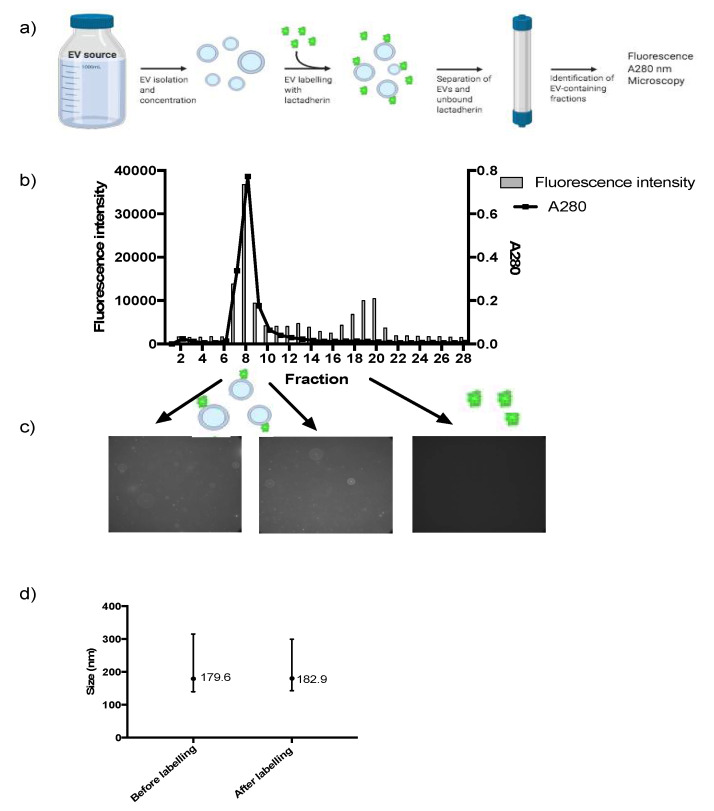
Labelling and identification of labelled extracellular vesicles (EVs). The workflow for labelling the EVs is shown in (**a**), (**b**) shows representative data of the total fluorescence in each fraction after separating EVs and unbound lactadherin using SEC. Two nicely separated peaks could be identified. The fractions containing EVs were spectrophotometrically determined by measuring absorbance at 280 nm, where EVs scatter light. Afterwards, fractions containing labelled EVs were pooled and used for experiments. (**c**) Confocal fluorescence microscopy confirmed the presence of EVs labelled with Alexa Fluor-conjugated lactadherin bound to in the fractions 7–9 identified from (**b**) and the absence of EVs in fraction 18–20 (100× magnification). (**d**) Particle size distribution was determined using the Nanoparticle Tracking Analysis on EVs after purification from milk and on labelled EVs after concentration and incubation with lactadherin. Data show the D10, mode (•) and D90 mean sizes. N = 5. Values are means ± SD.

Labelling was carried out by mixing the isolated and concentrated EVs with 200 nM Alexa Fluor-conjugated lactadherin to enable lactadherin binding to phosphatidylserine. Afterwards, separation of labelled EVs and unbound lactadherin was performed using SEC. Figure 1a sums up the labelling method. On the resulting SEC fractions, fluorescence and absorbance at 280 nm were measured to identify the fractions containing labelled EVs and unbound lactadherin (Figure 1b). A clear separation was achieved, which was further confirmed by analyzing the fluorescence containing fractions by confocal fluorescence microscopy (Figure 1c). The results showed that labeled EVs were only present in the first peak of the chromatogram.

It was also investigated whether the labelling reaction affected the size of the EVs, e.g., by fusion or aggregation in the subsequent 24-h incubation step at 37 °C or concentration step. To evaluate this average EV sizes were determined on isolated EVs and freshly labelled EVs (Figure 1d). Size profiles did not indicate any fusion or aggregation after labelling. In total, an EV isolate was obtained and subsequently labelled with Alexa Fluor-conjugated lactadherin using a setup that effectively removed unbound lactadherin from EVs.

### 3.2. Comparison of the Efficacy of Different Wash Buffers in Washing off Non-Internalized EVs

**Figure 2:**
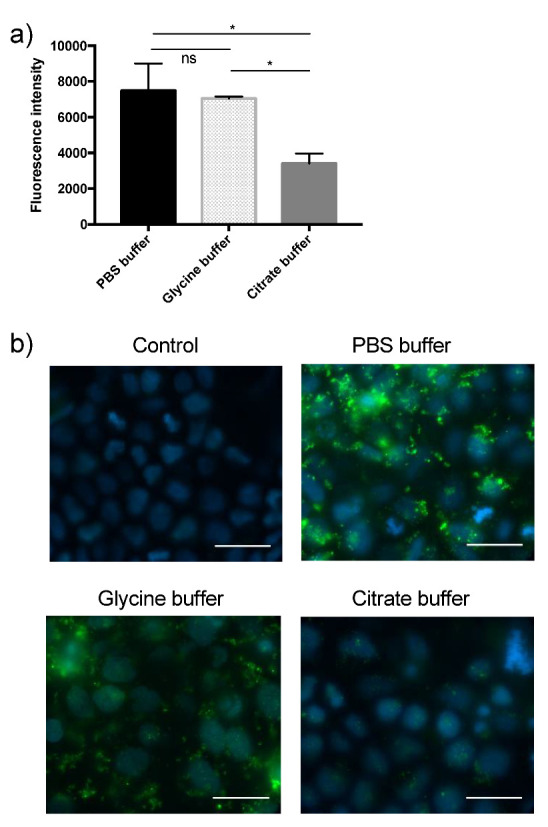
The importance of choosing an efficient buffer for cell washing. (**a**) Quantitative measurements of total fluorescence in cells after washing with the indicated buffer. The control is cells which did not incubate with labelled EVs and were washed in PBS. Control measurements are subtracted (N = 6.) Values are means ± SD. * statistically significant (*p* < 0.05), ns: not significant. (**b**) Confocal image of control cells after wash with test buffers. Test buffers were employed after stimulating Caco-2 cells with labelled EVs for two hours followed by a wash as described earlier. Scale bar 20 µm. Blue: 4′-6-diamidino-2-phenylindol (DAPI), green: EVs.

To be able to make reliable measurements of an EV uptake, different washing buffers were tested for their ability to remove EVs that was not taken up. Caco-2 cells were incubated with labelled EVs and successively washed three times with either of the described buffers. After washing, visual inspection was conducted to ensure that cell layers were intact. Finally, cells were lysed, and total fluorescence was measured. As seen from Figure 2a, fluorescence intensity varied between the three different washing buffers with highest fluorescence intensity in PBS buffer washed cells, followed by glycine buffer, and citrate buffer yielding the lowest measurements. These results clearly indicate that the washing buffers hold different capacities for removal of non-internalized EVs from the cell surface. Further investigations using widefield microscopy confirmed this, as EVs were clearly visible at the cell surface after washing with PBS buffer or glycine buffer, whereas citrate buffer proved more effective by resembling the control made without addition of labelled EVs (Figure 2b). For these reasons, citrate buffer was chosen as the preferred cell washing buffer in the remaining cell experiments.

### 3.3. Investigating EV Uptake and Cytotoxicity on Caco-2 Cells

**Figure 3:**
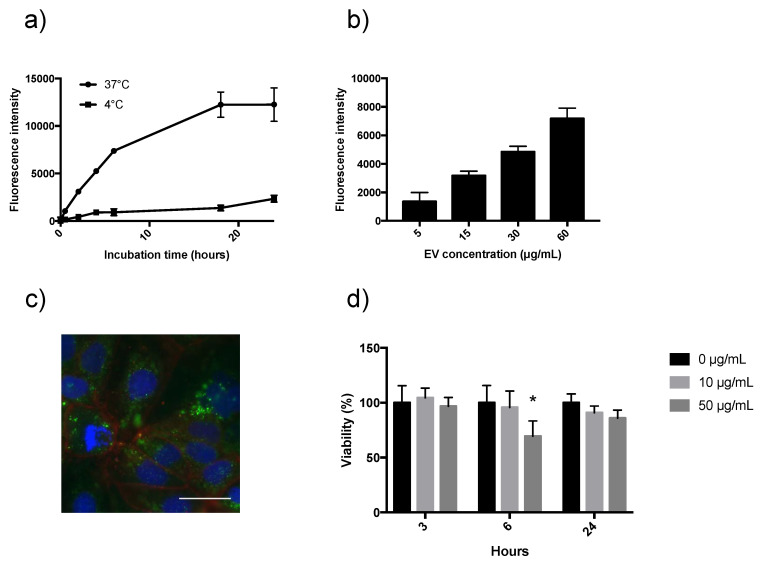
Fluorescently labelled EVs are taken up in vitro by intestinal epithelial cells. Caco-2 cells internalize EVs in an (**a**) time- and temperature-dependent manner (N = 5), and (**b**) in a dose-dependent manner after a four-hour incubation (N = 5). Control measurements (no EVs) are subtracted. (**c**) Uptake of fluorescently labelled EVs (green) after a four-hour incubation was imaged to visualize internalization; blue: DAPI, red: cell membranes Octadecyl Rhodamine B Chloride (R18), green: EVs. Scale bar 10 µm. (**d**) Cytotoxicity of EVs was evaluated using a MTT assay (N = 5). Caco-2 cells incubated with EVs at the indicated times (3, 6, or 24 h) and concentrations (0, 10, or 50 µg/mL). Values are means ± SD; * statistically different from control (0 µg/mL) (*p* < 0.05).

Next, the Alexa Fluor-labelled EVs and citrate buffer cell wash constituted the basis for evaluating in vitro cellular uptake. Initially, to address whether EV-uptake by Caco-2 cells is time-dependent, a longitudinal experiment was set up. Two cell experiments were run in parallel over 24 h; one incubating at 37 °C and the other at 4 °C. The result is shown in Figure 3a. An increased uptake over time was observed at 37 °C; however flattening after 18 h. Compared to EV uptake at 37 °C, incubation at 4 °C resulted in six-fold less fluorescence intensity after four hours, indicating that uptake is a temperature-dependent, active process. As suggested by the International Society of Extracellular Vesicles in a position letter from 2018, it was also of interest to characterize the uptake in a dose-dependent manner [42]. In cells incubated with 0, 5, 15, 30, or 60 µg protein/mL fluorescently labelled EVs for two hours, and from Figure 3b, it is strongly indicated that uptake is dose-dependent in the concentration range used in this experiment. EV uptake by Caco-2 cells was then visualized using fluorescent microscopy to confirm internalization (Figure 3c). An MTT assay was applied to address whether EVs displayed cytotoxicity towards the Caco-2 cells either with increasing concentration or incubation time. A typical example is presented in Figure 3d. Neither 10 µg/mL or 50 µg/mL caused cytotoxicity after 3 or 24 h when compared to cells not stimulated with EVs. Surprisingly, 50 µg/mL EVs significantly decreased cell viability after six hours, but this was not seen after 24 h. Successive experiments could not confirm this decrease of Caco-2 cell viability after six hours with 50 µg/mL EV.

### 3.4. Intracellular EV Sorting after Uptake

To further investigate EV uptake, cellular processing upon uptake in Caco-2 cells was addressed using co-localization studies. Rab5, Rab7, and Rab11 are Rab GTPases involved in intracellular vesicle transport and used as endosomal markers (see Table 1).

**Figure 4:**
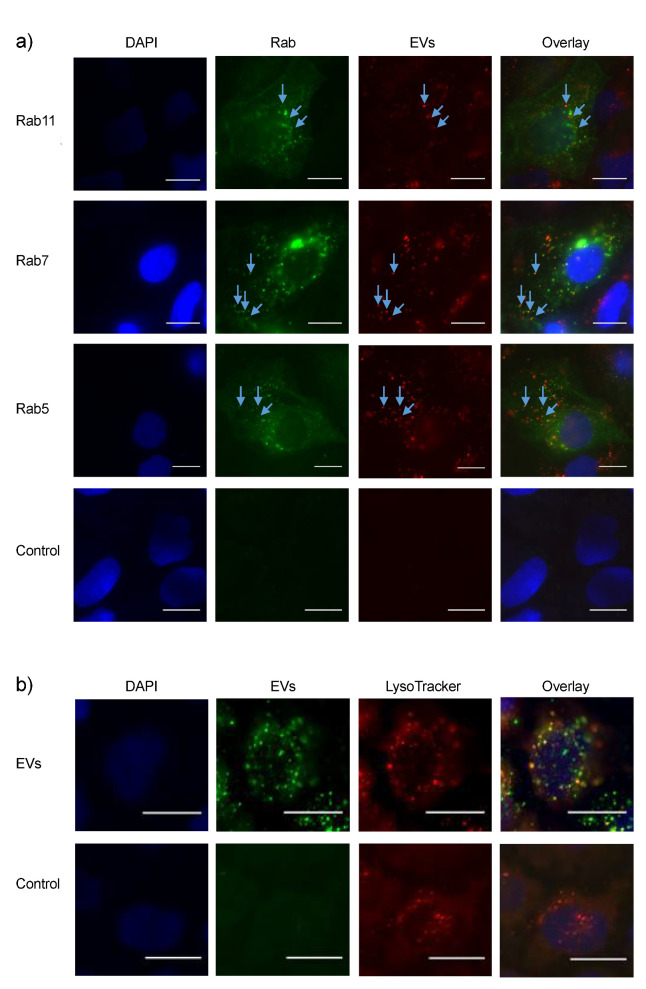
EVs are transported intracellularly via the endosomal system upon uptake. (**a**) EVs colocalize with early endosomes (Rab5), late endosomes (Rab7), and recycling endosomes (Rab 11), as indicated by the blue arrows on the overlaid images. The control has no EVs added. Scale bar 10 µm. (**b**) EVs also colocalize with lysosomes (labelled with LysoTracker), indicating that degradation of material occurs. Representative images were chosen for analysis. Incubation time with labelled EVs was four hours. Scale bar 10 µm.

The cells were transfected with one of the markers and incubated with labelled EVs for four hours before fixation. Determination of colocalization is based on the visual appearance of yellow spots on the overlaid images. As shown in Figure 4a, EVs colocalize with both Rab5, Rab7, and Rab11. Endocytosed material enters the endosomes in the intracellular space. Some of this material may end up in the lysosomes for degradation. To assess sorting of EVs into lysosomes, colocalization with lysosomes was assessed. The resulting images are shown in Figure 4b. According to the overlaid image, several EVs and lysosomes colocalize after four hours of incubation, indicating that EV material end up in lysosomes.

## 4. Discussion

A growing body of evidence suggests that EVs are communicative mediators, which shuttle extracellular messages from tissues within an organism. Interindividual communication via EVs is also possible via exchange of body fluids. EVs are abundantly present in raw milk, where they most likely deliver messages from the breastfeeding mother to her baby resulting in communication between individuals of same species. Milk EVs suggestively also convey cross-species communication when humans consume raw or processed milk from e.g., cows and goats.

The conducted experiments provide evidence that EVs from bovine milk are taken up by the human intestinal epithelial cell line Caco-2 cells in a dose-, temperature-, and time-dependent manner. By colocalization studies with endosomal markers for early, late, and recycling endosomes, it was demonstrated that the EVs are sorted intracellularly upon uptake and colocalize with all three endosomal markers. EVs also colocalize with lysosomes indicating that some EV-material is subject to lysosomal degradation.

### 4.1. Milk EV Isolation and Labelling

The EV isolation procedure described here, originally presented in [7], yields a EV pool well-separated from other milk constituents in a gentle way. However, it reflects the complexity of milk, by displaying some heterogeneity in composition and size in accordance with the generally accepted knowledge about the milk EV pool [6,7,39]. The labelling procedure was carried out on the isolated EV fraction using lactadherin conjugated to the fluorophore Alexa Fluor. Lactadherin has a strong affinity for phosphatidylserine (PS) exposed on the outer leaflet of phospholipid membranes and is already naturally present in the EV isolate. Although lactadherin is already present in the EV membrane, no limitations of the binding capacity were seen under the used conditions. The ability to attach largely all of administrated amount of labelled protein to the EVs might be explained by a dynamic exchange between intrinsic and added lactadherin. Lactadherin is very firmly attached to PS-exposing phospholipid membranes [39], and this might be the reason incubation for 24 h at 37 °C is needed to reach a satisfactory incorporation of the labelled protein. The labelling method presented here is a specific, yet time-consuming and laborious method. It labels PS-positive EVs, which eliminates the non-specificity of labelling with generic fluorescent dyes. However, PS-positive EVs might only constitute subfractions of the used EVs, as it has been reported that only a minority of blood plasma EVs expose PS [46]. Whether this is the case for milk EVs is not known, but lactadherin is indeed a valid marker for milk EVs [7].

A pilot study to stain isolated EVs with the lipophilic dye Octadecyl Rhodamine B Chloride (R18) was conducted; however excess dye was not separated from labelled EVs when applying SEC. Issues concerning separation of free dye from labelled EVs described elsewhere seem to be overcome with the labelling technique described here. Application of SEC yields an effective separation of EVs and free lactadherin, and the conducted fluorescence microscopy experiments document that labelled lactadherin end up in the EV-fraction as well (Figure 1c). The used technology is very gentle and might be preferred instead of separation by ultracentrifugation, as the latter might involve use of undesirable shear force. Furthermore, difficulties have been reported in the resuspension of EV pellets generated in attempts to separate labelled EVs from excess dye [6]. It is imaginable that the 24 h, at 37 °C, incubation period with labelled lactadherin could increase the risk for EV clogging or fusion. However, the average size of the EV population did not change after labelling (Figure 1d), nor did the number of particles, which highlights the delicate separation on SEC and indicates that clogging failed to happen.

### 4.2. Cellular Uptake and Intracellular Processing of EVs

To test whether milk EVs are taken up in vitro by intestinal epithelial cells, a range of optimizing experiments were carried out to ensure an efficient removal of non-internalized EVs from the plasma membranes. PBS buffer seems to be the preferred choice of washing buffer when searching the literature. The current results nicely demonstrate and visualize that citrate buffer is superior compared to PBS buffer and glycine buffer, which both failed to wash away non-internalized, membrane-attached EVs (Figure 2a,b). Additionally, a pilot study with phosphate buffer supplemented with acetic acid (0.2 M) and NaCl (0.5 M) was tested for its capacity to wash away EVs, but it was equally insufficient as glycine buffer. In line with the observations that EVs are difficult to remove from plasma membranes, other researchers have concluded that citrate buffer was superior to PBS in removing non-internalised EVs from the plasma membrane [27]. Citrate is well known for its ability to chelate divalent metal ions. Integrin function highly depends on different divalent cations, and integrins are present on both the surface of EVs and many cells to most likely facilitate binding [23].

In the present study, EV uptake by Caco-2 cells was investigated as a function of EV concentration or incubation time and temperature. As Figure 3a,b show, it was found that uptake increases with both increasing EV concentration as well as time of incubation. However, a steady state seemed to occur after 18 h, either reflecting a saturation of uptake or an intracellular processing of EVs equal to the uptake. A saturation of milk EV uptake as a function of concentration was also reported by [21], who found this saturation to be more pronounced in rat epithelial IEC-6 cells compared to the human colon cell line Caco-2. It cannot be excluded that there might be species-specific and cell line-specific differences in EV uptake, underlining that it is relevant to describe uptake kinetics for each cell line. The EV uptake was found to be dose-dependent, which also indicates dose-dependent functionalities of EVs. Further investigations may be conducted in a context where realistic milk EV concentrations are taken into account.

Cytotoxicity as a consequence of EV uptake was evaluated by an MTT assay, which is a colorimetric assay for assessing cell metabolic activity. Figure 3d shows how a 24 h incubation with EVs did not affect cell viability compared to control cells receiving a similar amount of EV-free PBS in the cell media. As such, this indicates that EVs neither positively nor negatively affect the metabolic activity of Caco-2 cells after 24 h of incubation. As cell viability is compared to control cells incubating the same amount of time, it is, however, not known if the cell viability in general is lower after 24 h compared to a shorter experimental time. The finding that EVs have no effect on cell viability after 24 h matches a comparable previous finding [47]. In that study, no change was reported in macrophage cell viability after incubation with milk EV in doses up to 200 µg protein/mL.

By using fluorescence microscopy to perform qualitative colocalization experiments, this report demonstrates how EVs colocalize with both early, late, and recycling endosomal compartments upon internalization (Figure 4a). These findings are in line with similar studies, where EVs are transported in endosomes to the nucleus [33] or to the ER [34]. Endosomal transportation is a result of EVs actively taken up by endocytosis, which is supported by the fact that EV uptake was reduced at 4 °C (Figure 3a).

It is interesting that EV material seems to be included in secreted cargo from the cells, as colocalization with recycling endosomes indicates. Moreover, lysosomal degradation is also a fate for EV material as seen from Figure 4b, where colocalization between the lysosomal marker LysoTracker and EVs is observed. However, it cannot be ruled out that a fraction of the enriched EV pool is entering the cells by other routes, e.g., fusion with the plasma membrane to release content directly to the cytosol. Additionally, the broad and heterogeneous EV pool obtained from milk might have different target cells and functions resulting in a range of intracellular fates upon uptake. It should moreover be emphasized/underlined that only the intracellular journey of lactadherin is tracked. Whether the EVs are intact or subject to degradation is not assessed with this approach. It is, therefore, recommendable to perform a dual labelling with at least two different components to clarify this matter.

In conclusion, the conducted experimental study contributes to the EV field with a new approach to fluorescently label isolated EVs. The label is conjugated to the protein lactadherin, which already qualifies as a fundamental EV marker. Using this labelling method, it is possible to avoid use of more generic fluorescent dyes, by which some undesirable side effects might follow. Moreover, it is revealed that citrate buffer is a better choice for removal of non-internalized EVs from outer cell membranes when assessing EV internalization in vitro. Fluorescently labelled bovine milk EVs were used to describe uptake by the intestinal epithelial cell line Caco-2, which internalize EVs in a dose- and time dependent manner. Colocalization studies show that EVs upon uptake are sorted intracellularly via the endosomal system and end up in either recycling endosomes or lysosomes for degradation.

## Figures and Tables

**Table 1:** Overview of the plasmid constructs used for colocalization experiments.

Construct	Organelle Marker
Rab5-GFP	Early endosomes
Rab7-GFP	Late endosomes
Rab11-mCherry	Recycling endosomes

## Supplementary Materials

The following are available online at https://www.mdpi.com/2227-9059/8/7/211/s1, Figure S1: Separation of EVs from major skim milk proteins.

## References

[1] Valadi H., Ekstrom K., Bossios A., Sjostrand M., Lee J.J., Lotvall J.O. (2007). Exosome-mediated transfer of mRNAs and microRNAs is a novel mechanism of genetic exchange between cells. Nat. Cell Biol..

[2] Pullan J.E., Confeld M.I., Osborn J.K., Kim J., Sarkar K., Mallik S. (2019). Exosomes as Drug Carriers for Cancer Therapy. Mol. Pharm..

[3] Andreas N.J., Kampmann B., Le-Doare K.M. (2015). Human breast milk: A review on its composition and bioactivity. Early Hum. Dev..

[4] Admyre C., Johansson S.M., Qazi K.R., Filen J.J., Lahesmaa R., Norman M., Neve E.P., Scheynius A., Gabrielsson S. (2007). Exosomes with immune modulatory features are present in human breast milk. J. Immunol..

[5] Lässer C., Alikhani V.S., Ekstrom K., Eldh M., Parede P.T., Bossios A., Sjostrand M., Gabrielsson S., Lotvall J., Valadi H. (2011). Human saliva, plasma and breast milk exosomes contain RNA: Uptake by macrophages. J. Transl. Med..

[6] Zonneveld M.I., Brisson A.R., van Herwijnen M.J., Tan S., van de Lest C.H., Redegeld F.A., Garssen J., Wauben M.H., Nolte-’t Hoen E.N. (2014). Recovery of extracellular vesicles from human breast milk is influenced by sample collection and vesicle isolation procedures. J. Extracell. Vesicles.

[7] Blans K., Hansen M.S., Sorensen L.V., Hvam M.L., Howard K.A., Moller A., Wiking L., Larsen L.B., Rasmussen J.T. (2017). Pellet-free isolation of human and bovine milk extracellular vesicles by size-exclusion chromatography. J. Extracell. Vesicles.

[8] Reinhardt T.A., Lippolis J.D., Nonnecke B.J., Sacco R.E. (2012). Bovine milk exosome proteome. J. Proteom..

[9] Hata T., Murakami K., Nakatani H., Yamamoto Y., Matsuda T., Aoki N. (2010). Isolation of bovine milk-derived microvesicles carrying mRNAs and microRNAs. Biochem. Biophys. Res. Commun..

[10] Quan S., Nan X., Wang K., Jiang L., Yao J., Xiong B. (2020). Characterization of Sheep Milk Extracellular Vesicle-miRNA by Sequencing and Comparison with Cow Milk. Animals.

[11] Ibrahim H.M., Mohammed-Geba K., Tawfic A.A., El-Magd M.A. (2019). Camel milk exosomes modulate cyclophosphamide-induced oxidative stress and immuno-toxicity in rats. Food Funct..

[12] Chen T., Xie M.-Y., Sun J.-J., Zhang Y.-L. (2014). Exploration of microRNAs in porcine milk exosomes. BMC Genom..

[13] Benmoussa A., Ly S., Shan S.T., Laugier J., Boilard E., Gilbert C., Provost P. (2017). A subset of extracellular vesicles carries the bulk of microRNAs in commercial dairy cow’s milk. J. Extracell. Vesicles.

[14] Naslund T.I., Paquin-Proulx D., Paredes P.T., Vallhov H., Sandberg J.K., Gabrielsson S. (2014). Exosomes from breast milk inhibit HIV-1 infection of dendritic cells and subsequent viral transfer to CD4+ T cells. Aids.

[15] Torregrosa Paredes P., Gutzeit C., Johansson S., Admyre C., Stenius F., Alm J., Scheynius A., Gabrielsson S. (2014). Differences in exosome populations in human breast milk in relation to allergic sensitization and lifestyle. Allergy.

[16] Sun Q., Chen X., Yu J., Zen K., Zhang C.-Y., Li L. (2013). Immune modulatory function of abundant immune-related microRNAs in microvesicles from bovine colostrum. Protein Cell.

[17] Izumi H., Tsuda M., Sato Y., Kosaka N., Ochiya T., Iwamoto H., Namba K., Takeda Y. (2015). Bovine milk exosomes contain microRNA and mRNA and are taken up by human macrophages. J. Dairy Sci..

[18] Munagala R., Aqil F., Jeyabalan J., Gupta R.C. (2016). Bovine milk-derived exosomes for drug delivery. Cancer Lett..

[19] Kusuma R.J., Manca S., Friemel T., Sukreet S., Nguyen C., Zempleni J. (2016). Human vascular endothelial cells transport foreign exosomes from cow’s milk by endocytosis. Am. J. Physiol. Cell Physiol..

[20] Carobolante G., Mantaj J., Ferrari E., Vllasaliu D. (2020). Cow Milk and Intestinal Epithelial Cell-derived Extracellular Vesicles as Systems for Enhancing Oral Drug Delivery. Pharmaceutics.

[21] Wolf T., Baier S.R., Zempleni J. (2015). The Intestinal Transport of Bovine Milk Exosomes Is Mediated by Endocytosis in Human Colon Carcinoma Caco-2 Cells and Rat Small Intestinal IEC-6 Cells. J. Nutr..

[22] Galley J.D., Besner G.E. (2020). The Therapeutic Potential of Breast Milk-Derived Extracellular Vesicles. Nutrients.

[23] Mathieu M., Martin-Jaular L., Lavieu G., Théry C. (2019). Specificities of secretion and uptake of exosomes and other extracellular vesicles for cell-to-cell communication. Nat. Cell Biol..

[24] Maas S.L.N., Breakefield X.O., Weaver A.M. (2017). Extracellular Vesicles: Unique Intercellular Delivery Vehicles. Trends Cell Biol..

[25] Mulcahy L.A., Pink R.C., Carter D.R. (2014). Routes and mechanisms of extracellular vesicle uptake. J. Extracell. Vesicles.

[26] Raposo G., Nijman H.W., Stoorvogel W., Liejendekker R., Harding C.V., Melief C.J., Geuze H.J.B. (1996). lymphocytes secrete antigen-presenting vesicles. J. Exp. Med..

[27] Feng D., Zhao W.-L., Ye Y.-Y., Bai X.-C., Liu R.-Q., Chang L.-F., Zhou Q., Sui S.-F. (2010). Cellular Internalization of Exosomes Occurs Through Phagocytosis. Traffic.

[28] Tian T., Wang Y., Wang H., Zhu Z., Xiao Z. (2010). Visualizing of the cellular uptake and intracellular trafficking of exosomes by live-cell microscopy. J. Cell. Biochem..

[29] Costa Verdera H., Gitz-Francois J.J., Schiffelers R.M., Vader P. (2017). Cellular uptake of extracellular vesicles is mediated by clathrin-independent endocytosis and macropinocytosis. J. Control. Release.

[30] Parolini I., Federici C., Raggi C., Lugini L., Palleschi S., De Milito A., Coscia C., Iessi E., Logozzi M., Molinari A. (2009). Microenvironmental pH is a key factor for exosome traffic in tumor cells. J. Biol. Chem..

[31] Caponnetto F., Manini I., Skrap M., Palmai-Pallag T., Di Loreto C., Beltrami A.P., Cesselli D., Ferrari E. (2017). Size-dependent cellular uptake of exosomes. Nanomedicine.

[32] Tian T., Zhu Y.L., Hu F.H., Wang Y.Y., Huang N.P., Xiao Z.D. (2013). Dynamics of exosome internalization and trafficking. J. Cell. Physiol..

[33] Santos M.F., Rappa G., Karbanova J., Kurth T., Corbeil D., Lorico A. (2018). VAMP-associated protein-A and oxysterol-binding protein–related protein 3 promote the entry of late endosomes into the nucleoplasmic reticulum. J. Biol. Chem..

[34] Heusermann W., Hean J., Trojer D., Steib E., Von Bueren S., Graff-Meyer A., Genoud C., Martin K., Pizzato N., Voshol J. (2016). Exosomes surf on filopodia to enter cells at endocytic hot spots, traffic within endosomes, and are targeted to the ER. J. Cell Biol..

[35] Morales-Kastresana A., Telford B., Musich T.A., McKinnon K., Clayborne C., Braig Z., Rosner A., Demberg T., Watson D.C., Karpova T.S. (2017). Labeling Extracellular Vesicles for Nanoscale Flow Cytometry. Sci. Rep..

[36] Simonsen J.B. (2019). Pitfalls associated with lipophilic fluorophore staining of extracellular vesicles for uptake studies. J. Extracell. Vesicles.

[37] Takahashi Y., Nishikawa M., Shinotsuka H., Matsui Y., Ohara S., Imai T., Takakura Y. (2013). Visualization and in vivo tracking of the exosomes of murine melanoma B16-BL6 cells in mice after intravenous injection. J. Biotechnol..

[38] Hvarregaard J., Andersen M.H., Berglund L., Rasmussen J.T., Petersen T.E. (1996). Characterization of glycoprotein PAS-6/7 from membranes of bovine milk fat globules. Eur. J. Biochem..

[39] Otzen D.E., Blans K., Wang H., Gilbert G.E., Rasmussen J.T. (2012). Lactadherin binds to phosphatidylserine-containing vesicles in a two-step mechanism sensitive to vesicle size and composition. Biochim. Biophys. Acta (BBA) Biomembr..

[40] Lötvall J., Hill A.F., Hochberg F., Buzás E.I., Di Vizio D., Gardiner C., Gho Y.S., Kurochkin I.V., Mathivanan S., Quesenberry P. (2014). Minimal experimental requirements for definition of extracellular vesicles and their functions: A position statement from the International Society for Extracellular Vesicles. J. Extracell. Vesicles.

[41] Thery C., Regnault A., Garin J., Wolfers J., Zitvogel L., Ricciardi-Castagnoli P., Raposo G., Amigoren S. (1999). Molecular characterization of dendritic cell-derived exosomes. Selective accumulation of the heat shock protein hsc73. J. Cell. Biol..

[42] Théry C., Witwer K.W., Aikawa E., Alcaraz M.J., Anderson J.D., Andriantsitohaina R., Antoniou A., Arab T., Archer F., Atkin-Smith G.K. (2018). Minimal information for studies of extracellular vesicles 2018 (MISEV2018): A position statement of the International Society for Extracellular Vesicles and update of the MISEV2014 guidelines. J. Extracell. Vesicles.

[43] Lowry O.H., Rosebrough N.J., Farr A.L., Randall R.J. (1951). Protein measurement with the Folin phenol reagent. J. Biol. Chem..

[44] Schacterle G.R., Pollack R.L. (1973). A simplified method for the quantitative assay of small amounts of protein in biologic material. Anal. Biochem..

[45] Schindelin J., Arganda-Carreras I., Frise E., Kaynig V., Longair M., Pietzsch T., Preibisch S., Rueden C., Saalfeld S., Schmid B. (2012). Fiji: An open-source platform for biological-image analysis. Nat. Methods.

[46] Arraud N., Linares R., Tan S., Gounou C., Pasquet J.M., Mornet S., Brisson A.R. (2014). Extracellular vesicles from blood plasma: Determination of their morphology, size, phenotype and concentration. J. Thromb. Haemost..

[47] Somiya M., Yoshioka Y., Ochiya T. (2018). Biocompatibility of highly purified bovine milk-derived extracellular vesicles. J. Extracell. Vesicles.

