# Supplementary material for: Specific and Non-Invasive Fluorescent Labelling of Extracellular Vesicles for Evaluation of Intracellular Processing by Intestinal Epithelial Cells
Source: Biomedicines. 2020 Jul 14;8(7):211. doi: 10.3390/biomedicines8070211 (PMC7400383; doi:10.3390/biomedicines8070211)

Specific and non-invasive fluorescent labelling of extracellular vesicles for evaluation of intracellular processing by intestinal epithelial cells

Maria S. Hansen [1,*], Ida S. E. Gadegaard [1], Eva C. Arnspang [2,†], Kristine Blans [1], Lene N. Nejsum [2] and Jan T. Rasmussen [1,*]

[1] Department of Molecular Biology and Genetics, Aarhus University, 8000 Aarhus, Denmark; Ida-gadegaard@hotmail.com (I.S.E.G.); krbla@arlafoods.com (K.B.)

[2] Department of Clinical Medicine, Aarhus University, 8000 Aarhus, Denmark; arnspang@kbm.sdu.dk (E.C.A.); nejsum@clin.au.dk (L.N.N)

* Correspondence: msh@mbg.au.dk (M.S.H.); jatr@mbg.au.dk (J.T.R.)

† Present affiliation: Department of Chemical Engineering, Biotechnology and Environmental Technology, University of Southern Denmark, 5230 Odense, Denmark

Supplementary figure 1: Separation of EVs from major skim milk proteins. The SEC chromatogram in figure S1a) with numbers indicating fractions used for Western blots in figure S1b) against CD81 and lactadherin. CD81 was present in the fraction 1. Lactadherin (appears in two glycosylation variants, therefore a double band is detected) was primarily present in fraction 1, but appeared also in fraction 2 and 3. Fractions from the first and well separated peak were pooled and used for further EV experiments.

a)

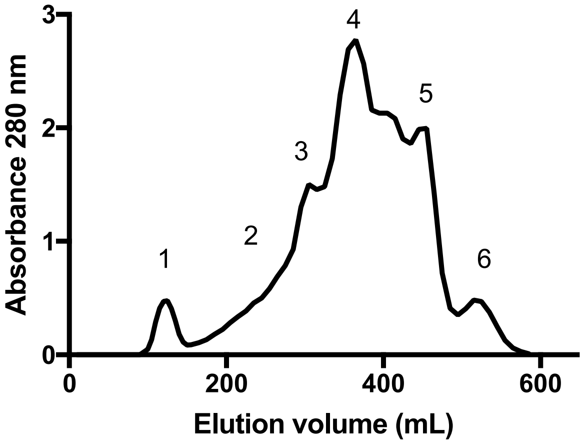

b)

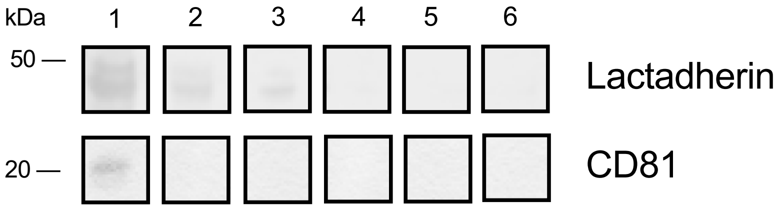

Supplement: Supplementary file 1 [file biomedicines-08-00211-s001.pdf]
